# Indirect and Unconscious Deception Detection: Too Soon to Give Up?

**DOI:** 10.3389/fpsyg.2021.601852

**Published:** 2021-05-26

**Authors:** Siegfried Ludwig Sporer, Joanna Ulatowska

**Affiliations:** ^1^Department of Psychology and Sports Science, University of Giessen, Giessen, Germany; ^2^Department of Psychology, Nicolaus Copernicus University, Toruń, Poland

**Keywords:** deception detection, direct lie detection, indirect lie detection, unconscious lie detection, veracity assessment, stimulus sampling, methodological recommendations for indirect and unconscious deception detection

## Introduction

In “direct” lie detection, the receiver of a message is explicitly asked to judge its veracity. In “indirect” or “implicit^1^ ” lie detection, the receiver is asked to rate some global impression(s) of the sender (e.g., “appears friendly,” “thinks hard”), sometimes with, sometimes without knowing that the study is about deception. “Unconscious” vs. “conscious” lie detection refers to the cognitive processes assumed to be involved in detecting deception. Some studies have shown that indirect or unconscious methods may lead to more accurate veracity assessment than a direct or conscious approach. However, results appear inconsistent, and it is unclear which mechanisms could be responsible for this advantage (Granhag, 2006). Some researchers suggest the role of unconscious knowledge or intuition (Reinhard et al., 2013a; ten Brinke et al., 2014,^2^). Others propose that indirect questions do not activate stereotypical beliefs about deception cues (Vrij et al., 2001; Street and Richardson, 2015).

Thus, although some of the veracity assessment methods labeled today as “indirect” or “unconscious” have been utilized for at least 40 years (DePaulo and Morris, 2004), most questions concerning their theoretical background, their accuracy, or practical applications (Granhag, 2006) remain unsolved. Even the simple decision to classify a procedure as “indirect” or “unconscious” still generates debate. This problem alone may be one of the reasons why conclusions from different studies on “indirect” or “unconscious” lie detection approaches appear so contradictory, leading Levine (2019) to question the value of these methods categorically.

It seems that different approaches have pursued their work from specific vantage points, ignoring each other like ships passing each other in the night. Although the five reasons Levine (2019) postulates are essential and well worth considering, his conclusions may be based on limited evidence [see Bond et al. (2015)]. Street and Vadillo (2016) have also criticized these approaches claiming the involvement of unconscious processes in lie detection as a specific instance of the general replication crisis observed with other social psychological phenomena attributed to unconscious processing. We concur with their theoretical arguments in so far as we also like to see evidence that indeed unconscious processes, in the absence of conscious processing, are at work in “unconscious lie detection.” However, we disagree with their conclusions based on Bond et al. (2015) meta-analysis (see below). Thus, we propose a broader view on the vast realm of studies and suggest that before researchers draw firm conclusions about these methods' potential, they should consider related theoretical approaches and their findings. To demonstrate the diversity of the procedures of studies using indirect or unconscious lie detection, we provide a detailed summary of the methodological aspects of these studies in Table 1. Furthermore, we draw attention to boundary conditions and moderator variables that should allow more differentiated conclusions about indirect and unconscious lie detection if considered in future research.

**Table 1:** Review of experiments on direct vs. indirect lie detection and unconscious vs. conscious lie detection.

**References**	**Exp**	**N Ps**	**N Stimuli (n Lies + n Truths)**	**Topic**	**Mode**	**Par**	**Aware**	**Design**	**Timing and form of veracity judgment**	**Indirect cues**
**Section I. Indirect vs. direct lie detection studies**										
DePaulo et al. (1982a)		176	48	E	A, AV	Ind	Yes	W	BT, (1–9 scale)	2 cues (1–9 scale): liking (?), mixed feelings (−1)
DePaulo et al. (1982b)		80	20 (10 + 10) ^*^ 2 sets	E	A, AV, T, ToV, V	Ind	Yes	W	BT, (1–9 scale)	6 cues (1–9 scale): liking (?), ambivalence (−1), disliking (?), discrepancy (−1), tension (−1), indifference (−1)
Anderson et al. (1999)		26	8 (4 + 4)	F	L	Ind	Yes	W	B, (0/1)	Open-ended answers coded into 16 cues: complexity (?), factual consistency (?), demeanor (?), involvement (?), flow (?), hunch (?), plausibility (?), restatement (?), sensitivity (?), verbal/nonverbal consistency (?), logic (?), would have told (?), verbal (?), paralinguistic (?), visual (?), other (?); And then to 3 cue composites: verbal/ consistency/ plausibility (?), paralinguistic/ flow (?), visual/ demeanor (?)
Vrij et al. (2001)		39	14 (7 + 7) ^*^ 2 sets	F	AV	Ind	No	B	(1–7 scale)	1 cue (1–7 scale): thinking hard (−1)
Landström et al. (2005)		116	1 of 14 (6 + 8)	F	AV, L	Ind	Yes	W	BT, lying (1–6 scale) + honesty (1–10 scale) + veracity (0/1) + confidence (50%-100% scale)	15 cues (1–7 scale): plausible (+1), detailed (+1), convincing (+1), confident (+1), think hard to remember (−1), defensive (?), involved (?), sympathetic (?), natural (?), active (?), straightforward (?), eloquent (?), relaxed (?), pleasant (+1), forthcoming (+) + 3 questions on memory self-assessment (1–7 scale) + memory accuracy
Landström et al. (2007)		122	1 of 12 (6 + 6)	F	AV, L	Ind	Yes	W	BT, (0/1) + justification	14 cues (1–10 scale): convincing (+1), confident (+1), detailed (+1), forthcoming (+1), plausible (+1), pleasant (+1), think hard to remember (−1), involved (?), natural (?), active (?), straightforward (?), eloquent (?), relaxed (?), defensive (?), + 4 questions on memory self-assessment (1–10 scales) + memory accuracy
Hart et al. (2009)		104	20 (10 + 10)	F	AV	Ind	No	B	(0/1) + confidence (1–7 scale)	1 cue (0/1): change in behavior, body language, or speech (−1) + confidence (1–7 scale)
Klaver et al. (2009)		444	2 (1 + 1) ^*^ 36 sets	F	AV	Ind	Yes	W	A, (0/1: pick which clip of 2 was true) + credibility (0–4 scale) + confidence (0–4 scale)	4 cues (0–4 scale): thinking hard (−1), nervous/fidgety (−1), emotional arousal (−1), attempting to control behavior (−1)
Stel et al. (2009)		46	1 of 46 (23 + 23)	F	L	Ind	No	W	B (1–7 scale)	12 cues (1–7 scale): frightened (−1), fearful (−1), anxious (−1), nervous (−1), penitential (−1), regretful (−1), guilty (−1), repentant (−1), enthusiastic (?), pleased (?), cheerful (?), happy (?)
Landström and Granhag (2010)		240	1 of 18 (9 + 9) ^*^ 3 sets	F	AV, CCTV, L	Ind	Yes	W	BT, (0/1) + which type of information was used to justify veracity assessment: nonverbal or verbal (1–7 scale)	13 cues (1–7 scale): detailed (+1), plausible (+1), convincing (+1), confident (+1), involved (?), nervous (−1), straightforward (?), natural (?), eloquent (?), forthcoming (+), pleasant (+1), think hard to remember (−1), defensive (?)
Ulatowska (2010)		101	8 (4 + 4) ^*^ 2 sets	E	AV	Ind	Varied as IV	B	(1–7 scale)	1 cue (1–7 scale): senders' confidence in their opinion (+1)
Bradford et al. (2013)		46	24 (12 + 12)	F	A, AV, T	Ind	Yes	W	B, (0/1)	4 cues (1–7 scale): confidence (+1), adequate information (+1), thinking hard (−1), suspiciousness (−1)
ten Brinke et al. (2014)	1	72	12 = 6 pairs (1 + 1)	F	AV	Ind	Yes	W	B (0/1)	IAT: Response latencies to faces of liars and truth-tellers (transformed to *d* scores)
	2	66	12 = 6 pairs: 2 unmatched (truth-lie) and 4 matched (2 truth-truth, 2 lie-lie)	F	AV	Ind	Yes	W	A (0/1)	IAT: Response latencies to faces of liars and truth-tellers (transformed to *d* scores)
Ulatowska (2014)	1	47	8 (4 + 4) ^*^ 2 sets	E	AV	Ind	Indirect + direct vs. indirect only	W	(1–9 scale)	6 cues (1–9 scale): sender's confidence (+1), thinking hard (−1), ambivalent emotions (−1), enough information (+1), feeling comfortable (+1), feeling suspicious (−1)
	2	122	8 (4 + 4) ^*^ 2 sets	E	AV	Ind	No	B	(1–9 scale)	3 cues (1–9 scale): sender's confidence (+1), thinking hard (−1), ambivalent emotions (−1)
van't Veer et al. (2015)		155	4 (2 + 2)	F	AV	Ind	No for first 2 videos; Yes for last 2 videos	W	A (0/1)	2 cues (1–7 scale): liking a sender (+1), trustworthiness (+1) + finger temperature
Canter et al. (2016)		30	12 (6 + 6)	F	AV	Ind	Yes	W	BT (1–5 scale): deceptive, genuine, trustworthy + truthfulness	10 cues (1–5 scale): open (+1), emotional (+1), facial pleasantness (+1), facial animation (+1), arousal (−1), tension (−1), involvement (−1), verbal consistency (+1), verbal plausibility (+1), verbal directness (+1), vocal certainty (+1)
Evanoff et al. (2016)		231	20 (10 + 10)	F	A, AV, T, V	Ind	Yes	W	B, (0/1)	7 cues (1–7 scales): happiness (?), sadness (?), fear (?), disgust (?), anger (?), surprise (?), sympathy (?)
Stel and Van Dijk (2018)	1	53	8 (4 + 4)	E	V	Ind	Yes	W	B, (1–7 scale)	12 cues (1–7 scale): tense (?), enthusiastic (?), pleased (?), worried (?), irritated (?), angry (?), confused (?), cheerful (?), dreary (?), happy (?), sad (?), mad (?). Then recoded to positive vs. negative emotions
	2	80	12 (6 + 6)	E	V	Ind	Yes	W	B, Truth (1–7 scale) + Lie (1–7 scale)	18 cues (1–7 scale): frightened (?), fearful(?), anxious (?), nervous (?), penitential (?), regretful (?), guilty (?), repentant (?), sad (?), angry (?), tense (?), worried (?), irritated (?), confused (?), enthusiastic (?), pleased (?), cheerful (?), happy (?). Then recoded to positive vs. negative emotions
Ulatowska (2018)	1	123	12 (6 + 6) ^*^ 2 sets	F	AV	Ind	No	B	(1–9 scale)	1 cue (1–9 scale): thinking hard (−1)
	2	33	12 (6 + 6)	F	AV	Ind	Yes	W	BT, (1–9 scale)	3 cues (1–9 scale): thinking hard (−1), did the interviewee actually plan to swap the answer sheets? (−1), is likely that the interviewee did actually swap the answer sheets (−1)
**Section Ia. Rapid judgments lie detection studies**										
Vrij et al. (2004)		5	52 (26 + 26)	F	AV	RapJ	Yes	W	A (0/1)	12 cues (1–5 scale; rapid judgments): hand/finger movements (+1), latency period (−1), speech hesitations (−1), quantity of details (+1), contextual embedding (+1), reproduction of conversation (+1), other's mental state (?), visual details (+1), auditory details (+1), spatial details (+1), temporal details (+1), cognitive operations (?)]
Dunbar et al. (2017)		8	73	F	AV	RapJ	Yes	W	A, (1–7 scale)	7 cues (1–7 scale; rapid judgments): quantity of details (+1), contextual embedding (+1), reproduction of conversation (+1), visual details (+1), auditory details (+1), temporal details (+1), spontaneous corrections (+1)
**Section Ib. The following studies are listed separately due to idiosyncrasies of design and methodology that make them incomparable to other indirect vs. direct studies**										
DePaulo and Rosenthal (1979)		40	120 ^*^ 2 sets	E	AV	Underlying affect detection	Yes	W	BT (1–9 scale)	5 cues (1–9 scale): liking (?), ambivalence (−1), disliking (?), discrepancy (−1), tension (−1)
Hurd and Noller (1988)		38	32 (16 + 16)	E	AV	Underlying affect detection	Yes	W	A (1–6 scale vs. 4FC format)	Think aloud protocol
Bond et al. (1990)		192	60 (30 + 30) ^*^ 2 sets	E	V	Underlying affect detection	Yes	W	A (0/1)	1 cue (binary): liking + 8 cues (independent raters): eye contact, smiling, head movements, blinking, self-touching hand gestures, unfilled pauses, filled pauses, negative terms + length of segment
Street and Richardson (2015)	1a	37	18 (9 + 9)	F	AV	Ind	No	N	N	2 cues (1–10 scale + 0/1; within-Ps): thinking hard (−1), tense (−1)
	1b	28	18 (9 + 9)	F	AV	Ind	No	N	N	2 cues (1–10 scale; within-Ps): thinking hard (−1), tense (−1)
	2	62	12 (6 + 6)	F	AV	Ind (not recorded)	Yes	W	A (0/1)	2 indirect (between-Ps, continuous rating, not recorded): thinking hard (−1), tense (−1)
**Section II. Unconscious vs. conscious lie detection studies**										
Feeley and Young (2000)		104	6 (3 + 3)	F	AV	Concurrent task	Yes	B	(0/1)	
Albrechtsen et al. (2009)	1	80	10 (5 + 5)	F	AV	Thin slices vs. full videos	Yes	B	(0/1)	
	2	120	10 (5 + 5)	F	AV	Concurrent task	Yes	B	(0/1)	
Reinhard et al. (2013a)	1	66	8 (4 + 4) ^*^ 2 sets	F	AV	Unc	Varied as IV	B	(0/1)	
	2	116	8 (4 + 4) ^*^ 4 sets	A	AV	Unc	Varied as IV	B	(0/1)	
	3	120	4 (2 + 2) ^*^ 5 sets	F	AV	Unc	Varied as IV	B	(0/1)	
	4	83	8 (4 + 4) ^*^ 4 sets	A	AV	Unc	Varied as IV	B	(0/1)	
	5	216	1 of 72 (36 + 36)	A	AV	Unc	Varied as IV	B	(0/1)	
Moi and Shanks (2015)	1	116	8 (4 + 4) ^*^ 2 sets	F	AV	Unc	No	B	(0/1)	
	2	110	8 (4 + 4) ^*^ 2 sets	F	AV	Unc	No	B	(0/1)	
Wu et al. (2019)	1	145	10 (5 + 5)	F	A, AV	Unc	Yes	B	(0/1)	
	2	140	10 (5 + 5)	F	AV, T	Unc	Yes	B	(0/1)	

*Although we did not aim to conduct a meta-analysis or a full systematic review, we carried out two thorough searches in August 2020 to identify publications on indirect and unconscious lie detection. The first search was carried out in the EBSCO database using relevant keywords “indirect,” “implicit,” “unconscious” AND “lie detection,” or “deception detection.” To identify additional studies on these topics, which for example, were conducted before these terms were in use, we searched references of review and meta-analytical articles and chapters on indirect and unconscious lie detection. We included only empirical articles and excluded studies where the rating of confidence of veracity judgment was the only indirect measure*.

*Exp, Experiment number; Ps, participants = judges/raters/coders; N Ps, number of judges/raters/coders; N Stimuli (n Lies and n Truths), the number of stimulus persons or accounts = senders telling a lie or a true account assessed by one participant*.

*Topic: Deception about: A, attitude; E, emotions; F, facts or action*.

*Mode: A, audio only; AV, audio-visual; L, live; T, transcript; ToV, tone of voice; V, video only*.

*Paradigm: Ind, indirect vs. direct; RapJ, rapid judgment; Unc, unconscious vs. conscious*.

*Aware, awareness of deception task: No vs. Yes vs. Varied as an independent variable*.

*Design: W, within-participants: Ps rate indirect cues and assess veracity; B, between-participants: rate indirect cues or assess veracity; N, no comparison*.

*Timing and Form of Veracity Judgment: B, before indirect; BT, between indirect; A, after indirect; N, no direct condition. Binary judgment (0/1) or Rating on Likert scale*.

*Indirect Cues: Rating of cues: Binary judgment (0/1) or Likert scale. (+1), Expected to be more frequent in truths (if explicitly stated in the text); (−1), Expected to be more frequent in lies (if explicitly stated in the text), (?), No direction was explicitly stated in the text*.

## A Comparison of Paradigms

First, it is important to consider whether indirect and unconscious veracity assessment methods^3^ activate similar processes and may be treated as examples of the same lie detection paradigm. We think they are not. As summarized in Section I of Table 1, in studies on indirect lie detection, an observer is asked to assess some aspects of a sender's statement or behavior other than veracity (e.g., whether a sender is “thinking hard^4^”) or is asked to evaluate their cognitions or emotions evoked by a sender (e.g., whether the receiver felt comfortable watching a sender; Granhag, 2006). Although some argue that these indirect questions lead to more accurate intuitive judgments, others suggest that this method's postulated higher accuracy relies on redirecting the focus of a detector's attention away from veracity *per se* but instead on a small set of cues (or even on a single cue) considered more diagnostic (Vrij et al., 2001; Street and Richardson, 2015).

In studies on unconscious lie detection (Section II of Table 1), the controlled, conscious processing of the sender's statement or behavior is disrupted or impeded, usually by an additional task. Some of these studies applied methodology taken from the unconscious-thought theory (UTT; Dijksterhuis and Nordgren, 2006). The UTT assumes two modes of thought: conscious and unconscious, which have different characteristics and should be used under different circumstances (Dijksterhuis and Nordgren, 2006). According to the UTT, people often use these modes inappropriately; for example, they try to solve complex problems (here, assess the veracity) by engaging in conscious thinking rather than unconscious processing. Unconscious thought, which automatically processes available data while one is consciously engaged in a different task, allows for more processing capacity to deal with complex information (i.e., behavioral cues to deception) and prevents stereotyping (i.e., relying on inaccurate stereotypical deception cues). As a result, unconscious (automatic) lie detectors are more accurate than conscious (controlled) thinkers because they can analyze and consider a higher number of diagnostic cues (Reinhard et al., 2013a).

Although indirect lie detection and UTT-based veracity assessment are thought to result in decisions based on more diagnostic behavioral cues, both approaches seem to achieve it by engaging different psychological processes (see Granhag, 2006). However, what we miss in almost all UTT-based studies is that there is no explicit, independent evidence for “unconscious processes” and their relationships to the judgments made^5^.

In some studies on unconscious decision-making, participants are told beforehand that deception is an issue; thus, they are aware of their task (Table 1, column “Aware”). In others, they learn about it only after seeing a videotape. In the latter procedure, raters, who are not aware that the study is concerned with detecting deception, may find the task not very motivating. In contrast, in many studies on indirect deception detection, participants usually watch videotapes and rate their global impression of one or several of the senders' behaviors. In between-participants designs, another group of raters assesses the veracity of each account. In within-participants designs, raters judge the veracity either immediately before or after rating each stimulus, or, alternatively, before or after a block of stimuli.

Although within-participant designs (see Table 1, column “Design”) may have statistical advantages, we note several methodological issues: (1) If indirect and direct measures are used together, they may mutually influence each other. A possible solution would be a pretest–posttest with a control group mixed-model design as in studies on training to detect deception (see Hauch et al., 2016), or even a Solomon four-group design (Campbell and Stanley, 1963). (2) If there are many (similar or long) videos shown *en bloc*, judges may forget who said what. Therefore, some studies have used only a few short (or partial) video clips. (3) If the speaker is reintroduced via a photograph, veracity judgments may be affected by the sender's perceived attractiveness or likeableness (Zebrowitz et al., 1996). (4) Using short video segments (“thin slices”), perhaps without tone, favors automatic, rapid judgments over conscious, deliberate analysis of the content of the message. (5) Using only a few stimuli violates the principle of stimulus sampling (see below).

## Focus on the Decision-Making Process vs. the Outcome

We do not deny that research has shown the superiority of unconscious over conscious or deliberate decisions in various fields other than lie detection. However, as demonstrated by the disproportion of studies in Section I and Section II of Table 1, the evidence for this claim in the lie detection field is rather scarce and equivocal and may largely depend on the methodology used. Concerning any type of decision-making, people seem to have little insight into the reasons why they make certain decisions (Nisbett and Wilson, 1977). Nonetheless, “fast and frugal” decisions are claimed to be often accurate (Gigerenzer et al., 1999). In the eyewitness literature, there is also evidence that fast decisions are a reliable cue for their accuracy and that having witnesses “think aloud” (Ericsson and Simon, 1993) while arriving at their decisions improve observers' evaluations of the accuracy of these decisions (Kaminski and Sporer, 2017). We suggest that think-aloud protocols and Brunswikian lens model analyses^6^ could also help us understand the process of indirect or unconscious deception detection (see Reinhard et al., 2013a).

## Methodological Issues and Recommendations^7^

### Specific Observable Behavior Ratings vs. Global Indirect Cues

Our summary in Table 1 shows that indirect questions concern a broad variety of sender behaviors, ranging from more specific to global nonverbal and paraverbal cues (e.g., hand/finger movements, response latencies, nervous), personality attributes (e.g., active, eloquent), emotions (e.g., cheerful, angry), a variety of verbal content cues (e.g., detailed), as well as inferred processes (e.g., thinking hard) that may influence both verbal and nonverbal behaviors. Verbal content cue ratings were also sometimes more general (e.g., consistency, plausibility) or referred to specified content qualities as used in research on criteria-based content analysis (e.g., reproduction of conversations) and within the reality monitoring approach (e.g., temporal details; see Sporer, 1997, 2004).

Although more thorough comparisons of the efficacy of different types of indirect questions are needed (see Ulatowska, 2014, 2020), we argue that indirect questions regarding verbal content cues should lead to higher accuracies because this approach enables the rater to find more diagnostic cues (Street and Richardson, 2015). We present evidence on the possible superiority of verbal content cues below.

Moreover, we urge researchers not only to ask for assessments of global impressions like “thinking hard” but also to collect additional data on specific, observable behaviors from independent raters and correlate these two sets of data. This would add more objective, operational definitions of indirect questions to the global impression ratings.

Importantly, significant differences in indirect measures do not imply that judges would actually use these differences to classify accounts correctly. An additional experimental group is necessary to investigate this, in which raters who rated specific behaviors or global impressions will subsequently assess each statement's veracity based on these ratings. This methodology is usually used in studies on training to detect deception (Hauch et al., 2016).

When comparisons between lies and truths on any dependent measures are analyzed, effect sizes (Cohen's *d*) with 95% CIs for *all* behaviors observed should be reported (not only for significant differences). Before conducting a study, power analyses should be performed, and non-significant differences should be accompanied by Bayes factor analyses (which are beyond this paper's scope).

### Defining Accuracy

Usually, recipients are asked to either make binary judgments (0 vs. 1) or assess veracity on continuous credibility rating scales (see Table 1, last two columns). To make studies comparable, dependent variables need to be defined and labeled consistently. When rating scales are used (for veracity judgments or other measures), results should be referred to as a difference between lies and truths rather than lie/truth detection accuracy. When binary judgments were made, sometimes researchers wrote about “lie detection,” which makes it unclear whether accurate classifications of *true* statements are included in this term. To avoid such ambiguities, accuracy of classifications of lies (lie detection accuracy) and of truths (truth detection accuracy) and overall accuracy (averaged across both) should be reported.

In everyday life, we expect most statements to be truthful. When the number of lies and truths in a study is not equal (as is likely to be the case in the real world), the proportions should be specified because accuracy will depend on expected and actual base rates of lies in a set of stimuli. Moreover, reporting both lie and truth accuracy is particularly important in areas where there are likely to be very high or very low base rates of lies or truths. Consequently, a method with high false alarm rates (i.e., classifying a truth-teller as a liar) would lead to large numbers of false accusations. In certain situations (e.g., airport security settings), lying base rates may be very low, leading to high false alarm rates. Hence, these methods may only be used as screening devices to be followed up by more thorough investigations to obtain further evidence.

Both lie accuracy and truth accuracy can be combined via signal detection theory, yielding measures of performance (*d', A'*) as well as a (presumably independent) measure of response bias (*C, beta, beta”*). As recently proposed by Smith and Leach (2019), signal detection models also allow us to use confidence in a lie–truth detection judgment to achieve even better discrimination^8^. When accuracies are reported for different confidence levels, the proportions of cases in each confidence bin should also be noted.

For all differences between methods groups, effect sizes with the direction of effect (Cohen's *d*, or *odds ratio*s, not partial eta^2^) should be reported (see Sporer et al., 2021). Accuracies calculated from automatic classification methods (e.g., with artificial intelligence algorithms) and from multivariate classification methods (e.g., multiple discriminant or logistic regression analyses, in particular when they were obtained without cross-validation) must not be compared with the accuracies of human raters (Sporer et al., 2021).

### Sample Sizes of Judges vs. Sample Size of Senders

Many studies have used relatively large samples of judges but only small samples of stimuli to be judged (often only 8 or 10; Table 1, Columns: *N* Ps and *N* Stimuli)^9^. Note that with 8 or 10 accounts, an individual judge will only be accurate above chance level if she or he classifies 7 or 8 of 8 (or 9 or 10 of 10) accounts correctly according to the binomial distribution. While some accuracy differences may become significant at the aggregate level with large numbers of judges, this does not imply that an individual judge will achieve accuracy above chance. Considering that there is much more variability across studies to be explained in sender detectability than in judge ability (Bond and DePaulo, 2008a,b), studies ought to use large, representative samples of stimuli. Therefore, we expect that differences in detection accuracy will be more likely detected if participants rate large numbers of stimuli (e.g., in several sessions) and different types of stimuli (e.g., different lie scenarios). Furthermore, if multiple rating scales are used, sample sizes of stimuli should be large enough to allow for factor analyses and the construction of theoretically meaningful subscales (reporting Cronbach's *alphas* and corrected item-total correlations for each subscale; Sporer et al., 2021).

Using small samples of stimuli violates the principle of stimulus sampling (Wells and Windschitl, 1999): Findings should hold not only across large numbers of participants but also for large numbers of stimuli from different situations and contexts. Applied to detecting deception, outcomes may vary for different types of lies. Studies on indirect methods have sometimes used lies about emotions (toward a person or attitude object), sometimes only “thin slices” of behavior (i.e., short video segments), but also reports about facts or events. Studies on unconscious detection were more likely about accounts of complex (autobiographical) events. These differences in stimuli used (see Table 1) may explain why some replication attempts may have failed and why results from indirect approaches and UTT studies cannot really be compared.

### Effects of Presentation Medium

Bond and DePaulo's (2006) meta-analysis showed the lowest detection accuracy in the visual-only format and the highest when statements were presented in audio or audiovisual form. In the visual-only modality, receivers may rely on stereotypical nonverbal cues (The Global Deception Research Team, 2006), most of which are non-diagnostic (see DePaulo et al., 2003; Sporer and Schwandt, 2006, 2007). Furthermore, senders in auditory-only communication settings may have less opportunity to strategically adjust or manipulate their demeanor (Burgoon et al., 2005) or gain from their attractiveness or likeableness. Another explanation could be that the availability of auditory information allows an analysis of verbal content cues, which are more diagnostic than nonverbal cues (e.g., Reinhard et al., 2013b; see below). However, the deception medium's influence was rarely explicitly tested in indirect and, especially, unconscious lie detection studies (see Table 1, “Mode” column). If these approaches indeed focus detectors' attention on diagnostic cues, we would expect that narrowing their attention to one communication channel may lead to even higher accuracy.

Furthermore, audiovisual communication may contain both non-diagnostic stereotypical nonverbal cues as well as diagnostic content cues. Processing both the visual information and the verbal content information may lead to an overload of information that is too difficult to process and integrate. This argument would imply that transcripts that allow only the use of verbal content cues should lead to higher accuracy than studies using only visual cues. On the other hand, if the UTT-based lie detection is supposed to work better in complex decision conditions, its advantage over conscious lie detection may be more apparent in full audiovisual deception.

### Diagnosticity of Verbal Content Cues vs. Nonverbal Cues

Criticisms of the indirect and UTT approaches have argued that there are no diagnostic cues, and therefore, it is not surprising that they do not yield better results than direct lie–truth judgments (either on binary or continuous credibility rating scales). In contrast, we argue that there are valid verbal content cues to deception that have not been considered in these criticisms (Levine and Bond, 2014; Bond et al., 2015; see also Luke, 2019). Verbal content cues have been primarily studied in accounts about autobiographical events, not about reported emotions, opinions, or attitudes.

These recent reanalyses of cues to deception were based on DePaulo et al.'s (2003) comprehensive meta-analysis of published and unpublished studies of 1,338 estimates of 158 cues and on Bond and DePaulo's (2006) meta-analysis of accuracy rates in over 200 studies. It is important to note that in DePaulo et al.'s (2003) ubiquitously cited meta-analysis, all but four studies were from the years 1970 to 1997 (two studies were from the 1920s, one from 1998 and 1999 each). This era was dominated by Zuckerman, Ekman, DePaulo, Burgoon, and colleagues' communication research on nonverbal and paraverbal cues to deception, mostly about emotions but less frequently about facts or events. Hence, by relying on outdated databases, the mentioned recent reanalyses barely took the dozens of studies on verbal content cues to deception on CBCA (Steller and Köhnken, 1989) and reality monitoring criteria (Sporer, 1997, 2004) into account. DePaulo et al.'s meta-analysis included only six studies reporting any CBCA criteria, only four studies reporting any RM criteria, and two other studies reporting one additional verbal content cue each. It is clear that, since then, many more studies on verbal content cues have become available^10^ that have not been considered in the debate on unconscious lie detection by previous commentators.

In the last 5 years, several (but still incomplete) meta-analyses on verbal content cues have been published, which reported substantially larger effect sizes than any previous meta-analyses and reanalyses, with positive *d* values indicating a stronger presence of cues in true accounts—Amado et al., 2015: 16 studies with children, mean *d* = 0.40; Amado et al., 2016: 46 studies with adults, mean *d* = 0.25; Hauch et al., 2015: 79 linguistic cues in 44 studies, mean *d* for the length of accounts = 0.25; Hauch et al., 2016: 30 studies, mean *d* for eight training studies with content cues, *d* = 0.73 vs. six studies with nonverbal cues, *d* = 0.24; Oberlader et al., 2016,^11^: 56 effect sizes; Vrij's, 2005: vote-counting of 37 studies). While DePaulo et al. (2003) had reported a *Mdn d* = 0.10 across all cues, these content-based meta-analyses yielded much larger mean effect sizes for verbal content cues. Thus, the conclusions drawn from the reanalyses mentioned above are in dire need to be updated with these newer data sets of effect sizes.

However, before becoming too optimistic about these larger effect sizes, we caution the reader to critically scrutinize all, both older and newer, meta-analyses because a large number of studies contained therein have been statistically underpowered, biased by selective reporting and other “questionable research practices” and publication bias (for a thorough discussion of these and other issues, see Luke, 2019).

The availability of valid verbal content cues also has implications for the unconscious–conscious debate. The proper use of valid verbal content cues requires intensive training to code them reliably (Köhnken, 2004; Hauch et al., 2016, 2017, recommends a 3-week training program). Reliability of coding is a prerequisite for the discriminative validity of these cues. Applying them to a text corpus (written or oral) requires a careful analysis of all details of a statement, either by reading a transcript (or listening to an audio/videotape) repeatedly or taking notes. Normally, this requires a conscious, analytic approach—but see the study by Vrij et al. (2004) who have shown that rapid judgments may also catch some of these cues, which might be useful as a preliminary screening.

### Complexity of Accounts

Studies often vary widely in the length of the accounts evaluated (measured in seconds or number of words). If the assumptions of UTT are correct, complexity appears as a prerequisite. Hence, to test its effectiveness, stimuli ought to have sufficient length and complexity. With complex events and consequently longer accounts, the burden on working memory is higher. Hence, receivers may only remember and use gist memory when distracted and verbatim memory when deliberating (Abadie et al., 2016). Materials (video- or audiotapes) ought to be also of sufficient resolution and auditory quality.

Furthermore, because senders' statements depend on the questions asked, questions should also be included if an interview was used. As we know from eyewitness testimony research from the beginning of the 20th century (Stern, 1904; see Sporer, 2008), an answer can only be properly understood when the question is also known. Regarding deception, there is a voluminous body of research on false confessions that became apparent when the questions asked in the police interrogations became available (e.g., Garrett, 2010).

### Response Latencies Regarding Lies About Story Elements

Deception is not all or none. We need to distinguish studies investigating answers to individual questions from those evaluating an account's overall truthfulness assessed by a final veracity judgment. In the psychophysiological literature (National Research Council, 2003), comparisons are made not between different accounts or stimulus persons or between liars and truth-tellers but between response options of individual items (like the alternatives in a multiple-forced-choice test). A frequently used measure is response latency as an indirect indicator of an association (for a historical review, see Antonelli and Sporer, in press). For example, in a recent meta-analysis of studies on the concealed information test and similar paradigms, participants responded considerably faster to true than false items (Suchotzki et al., 2017). However, the effects varied widely, and countermeasures reduced the effect.

## The Case for Conscious vs. Unconscious Decision-Making in Criminal Courts

Consider for a moment an example of lie detection in a most serious context, the evaluation of the credibility of a witness or a defendant in a criminal court case. Depending on the judiciary system, the decision about the determination of guilt lies in the hands of professional judges or a jury.

Leaving the issue of group vs. individual decision-making aside, judges or jurors must decide whether a witness (or a defendant) is telling the truth. For a procedure to be fair, would you not expect, or even demand, that fact finders pay close attention to the witness's utterances, including both the content and the “demeanor” with which the statement is provided?^12^ Would you not expect that any statements be critically scrutinized by consciously deliberating any nuances and possibilities indicating the possibility of deception? Would you consider it fair if a judge or jurors were not paying attention before arriving at a decision in the case?

When deciding on a sentence, judges must provide “reasons” why a particular decision was made, including a rational evaluation of the witness's incriminating evidence. This requirement led the German Supreme Court to overturn lower-court decisions (Bungesgerichtshof in Strafsachen (BGHSt), 1999), in which “experts” had insufficiently assessed key witness statements. As a consequence, the Supreme Court demanded that, in future cases, experts should follow a series of guidelines for Statement Validity Analysis and Criteria-Based Content Analysis (see Steller and Köhnken, 1989) and explicitly refer to them in their expert testimony. In our view, this would leave no room for unconscious assessments. In another case in Canada, the question arose whether a witness ought to remove her niqab while testifying for her demeanor to become visible (see Snook et al., 2017; Denault and Jupe, 2018). This is an interesting empirical issue demonstrating the importance of our discussion in the real world.

Of course, there is a long literature on judicial decision-making demonstrating that “unconscious” factors may determine such decisions (e.g., Goodman-Delahunty and Sporer, 2010). Consequently, both experts and courts may be affected by a host of biases amply documented in the social psychological literature (Pohl, 2004). We also know from that research that reasons to justify a decision are often *post-hoc* rationalizations of a previous decision to make it appear rational and protect judges from appellate courts overturning their decisions. Nonetheless, we would hope that decisions about defendants' lives are made with the uttermost diligence and careful (“conscious”) consideration of all facts pertinent to the decision. But just how these facts are integrated into a final decision remains a puzzle.

## Conclusion

Despite inconsistent results, we argue that indirect methods to veracity assessment deserve further attention for the following reasons: (1) Indirect measures and “unconscious” approaches should be pursued separately. (2) Critiques on indirect approaches were based on outdated meta-analytical databases summarizing mainly research on nonverbal and paraverbal cues and only a handful of verbal content cues. These databases need to be updated by newer studies and meta-analyses on verbal content cues with higher diagnostic value. (3) Research on detecting emotions and opinions has to be treated separately from assessing the veracity of complex reports about facts or events. (4) More emphasis should be placed on stimulus than on participant sampling. (5) The relationships and mutual dependencies of indirect cues and explicit veracity judgments need to be further explored. (6) Evidence for “unconscious” processes needs to be provided.

## Author Contributions

All authors have made substantial contributions to planning, writing and commenting the content, and also approved the version to be submitted in the journal.

## Conflict of Interest

The authors declare that the research was conducted in the absence of any commercial or financial relationships that could be construed as a potential conflict of interest.

## Footnotes

^1^Some authors have used the term “indirect,” others “implicit” (e.g., Granhag, 2006) but referring to similar methods. We generally use the term “indirect” except when referring to specific studies where we use the respective authors' term. Otherwise, we consider them interchangeable but distinct from “unconscious lie detection.”

^2^There is a severe criticism of the conclusions drawn from the statistics presented in this study by Franz and von Luxburg (2015) that is beyond the scope of this article.

^3^Apart from indirect or unconscious lie detection, some studies used rapid judgments or other ratings to assess verbal content like plausibility, detailedness, etc. (Table 1, Section 1a). We argue that this type of global impression judgment may be valuable as a screening device when deciding whether a detailed content analysis proposed in the SVA/CBCA or the Reality Monitoring approach (see below) may be useful. However, the latter methods require a thorough conscious analysis of a text corpus that is only possible after intensive training, with high demands on interrater reliability. These types of content analysis are not an indirect or unconscious process approach.

^4^As presented in Table 1, Section I, “thinking hard” is the most frequently used indirect measure (40% of studies), followed by a question about a sender's confidence (20% of studies).

^5^As far as we can tell, only Reinhard et al. (2013a, Exp. 5) (Table 1, Section II) made such an attempt. However, the pattern of objectively diagnostic and non-diagnostic cues in that study seems to reflect stereotypical cues and is also at odds with research on verbal content cues to deception (Amado et al., 2016) and the study on rapid judgments by Vrij et al. (2004).

^6^See Fiedler (1989), Köhnken (1990); Sporer and Küpper (1995), and Hartwig and Bond (2011) for detailed descriptions of the model in the lie detection domain.

^7^Some of these recommendations are suitable for all types of lie detection studies.

^8^Initially used in radar technology and further developed in medical and perception research, the SDT methodology here is borrowed from face recognition and eyewitness identification studies, where it has become standard (Macmillan and Creelman, 2005; Wixted and Wells, 2017).

^9^In Table 1, the total number of senders varied between 1 and 73 (*M* = 17.23; *SD* = 18.05) in studies on indirect measures (Section I and Ia) and between 1 and 10 (*M* = 7.58; *SD* = 2.74) in studies on unconscious lie detection (except one study that had a total of 72 senders but only one per participant) (Section II).

^10^The first author has over 100 studies on verbal content cues in his database.

^11^Note that this meta-analysis integrated effect sizes of mean differences of cue summary scores with effect sizes from accuracy rates of raters and accuracy rates based on multivariate statistical analyses. Because the latter were usually obtained without cross-validation, their integration with effect sizes based on accuracy rates of human raters is problematic (see Sporer et al., 2021). The same criticism holds for Vrij (2005, Table 4) averaging of rater judgment accuracies and accuracies from multivariate statistical analyses.

^12^In the communication literature, researchers have investigated for over half a century which aspects of “demeanor” (i.e., nonverbal and paraverbal cues) may reveal deception. In a widely received article by Denault and several dozen deception researchers (Denault et al., 2020), judicial, governmental, and security agencies are criticized for relying on pseudoscientific advisors (e.g., synergologists) instead of relying on proper, peer-reviewed science, costing the public millions of dollars. Police, attorneys, and judges often appear uninformed (or even unaware of its existence) about the evidence-based detection of deception literature (Jupe and Denault, 2019).
